# During Hospitalization, Older Patients at Risk for Malnutrition Consume <0.65 Grams of Protein per Kilogram Body Weight per Day

**DOI:** 10.1002/ncp.10542

**Published:** 2020-06-24

**Authors:** Michelle E. G. Weijzen, Imre W. K. Kouw, Phil Geerlings, Lex B. Verdijk, Luc J. C. van Loon

**Affiliations:** ^1^ Department of Human Biology School of Nutrition and Translational Research in Metabolism (NUTRIM), Maastricht University Medical Centre+ Maastricht the Netherlands; ^2^ Department of Dietetics Maastricht University Medical Centre+ Maastricht the Netherlands

**Keywords:** energy, hospitalization, malnutrition, oral nutrition supplements, older adults, protein

## Abstract

**Background:**

Malnutrition is prevalent in hospitalized patients. To support muscle maintenance in older and chronically ill patients, a protein intake of 1.2–1.5 g/kg/d has been recommended during hospitalization. We assessed daily protein intake levels and distribution in older patients at risk for malnutrition during hospitalization.

**Methods:**

In this prospective, observational study, we measured actual food and food supplement consumption in patients (*n* = 102; age, 68 ± 14 years; hospital stay, 14 [8–28] days) at risk of malnutrition during hospitalization. Food provided by hospital meals, ONS, and snacks and the actual amount of food (not) consumed were weighed and recorded for all patients.

**Results:**

Hospital meals provided 1.03 [0.77–1.26] protein, whereas actual protein consumption was only 0.65 [0.37–0.93] g/kg/d. Protein intake at breakfast, lunch, and dinner was 10 [6–15], 9 [5–14], and 13 [9–18] g, respectively. The use of ONS (*n* = 62) resulted in greater energy (1.26 [0.40–1.79] MJ/d, 300 [100–430] kcal/d) and protein intake levels (11 [4–16] g/d), without changing the macronutrient composition of the diet.

**Conclusion:**

Despite protein provision of ∼1.0 g/kg/d, protein intake remains well below these values (∼0.65 g/kg/d), as 30%–40% of the provided food and supplements is not consumed. Provision of ONS may increase energy and protein intake but does not change the macronutrient composition of the diet. Current nutrition strategies to achieve the recommended daily protein intake in older patients during their hospitalization are not as effective as generally assumed.

## Introduction

Hospitalization is accompanied by substantial changes in habitual food intake. Food intake during hospitalization is typically reduced because of restricted timing of food provision, adverse effects of medication, a reduced appetite, and prescribed periods of fasting.^1^, ^2^, ^3^ Malnutrition during hospitalization is a critical and highly prevalent problem, as up to 40% of the patients have been reported to be malnourished during their hospital stay.^4^, ^5^ Malnutrition is defined as a deficiency in energy, protein, and/or micronutrients^6^, ^7^ and is generally accompanied by a more adverse clinical outcome during hospital stay. During hospitalization, malnutrition in patients has been shown to prolong the length of stay (LOS),^4^, ^8^, ^9^ accelerate the loss of muscle mass,^10^ impair functional outcome,^11^ and increase the risk of morbidity and mortality.^8^, ^9^, ^12^

Accelerated loss of skeletal muscle mass and strength is, at least partly, attributed to the negative health consequences of malnutrition. Muscle mass loss during hospitalization results in functional decline and the loss of independence in older patients.^13^ Muscle atrophy, already observed during a few days of hospitalization,^14^, ^15^, ^16^ is accelerated by the associated physical inactivity and/or an insufficient protein intake as a direct consequence of the lower energy intake. The current recommended dietary intake for protein is set at 0.8 g/kg/d for healthy adults of all ages according to the World Health Organization (WHO).^17^ Recent European Society for Clinical Nutrition and Metabolism (ESPEN) nutrition guidelines recommend a higher protein intake level of 1.2–1.5 g/kg/d for malnourished patients or patients at risk for malnutrition due to acute or chronic illness, as a means to support muscle mass and strength maintenance.^18^, ^19^ Previously, we showed that during short‐term hospitalization, protein intake falls well below 0.8 g/kg/d in patients undergoing elective orthopedic surgery.^20^ In line with this finding, several other studies have reported that hospitalized patients do not meet these recommended protein intake levels.^21^, ^22^, ^23^ So far, most studies have assessed food intake by using estimated dietary intake records or food frequency questionnaires in hospitalized patients,^23^, ^24^, ^25^ whereas only a few studies have actually measured the amount of food that was consumed.^20^, ^21^, ^22^ A difference of 30%–40% between food provision and food consumption has been reported during hospital stay, resulting in low energy and protein intake levels in older patients.^20^, ^26^, ^27^, ^28^

In current practice, patients at risk for malnutrition are referred to a dietitian during hospitalization. Consequently, various nutrition strategies are applied to increase protein intake. The use of more protein‐rich foods in the diet,^29^ protein fortification of meals,^21^, ^30^ and/or the provision of oral nutritional supplements (ONS)^31^, ^32^, ^33^ are being applied to increase protein intake during hospitalization. However, there are currently limited data on actual protein consumption in older patients at risk of malnutrition, their protein intake distribution pattern, and the actual consumption of the prescribed ONS during hospitalization. In the present study, we therefore assessed both food provision and food consumption from self‐selected hospital meals and the actual intake of ONS and snacks in 102 older patients at risk of malnutrition during several days of hospital admission. We hypothesized that despite the existing nutrition strategies to increase protein intake, older patients at risk for malnutrition do not achieve current protein intake guidelines during hospitalization.

## Methods

### Study Design

In the current prospective observational study, the nutrition content of hospital meals, snacks, and ONS was assessed, and actual food, snack, and ONS consumption was measured in *n* = 102 patients during their hospitalization. Three different nursing wards (respiratory *n* = 36, geriatric *n* = 32, and general surgery *n* = 34) were selected to include patients between February 2017 and June 2017. The Malnutrition Universal Screening Tool (MUST) was used to screen patients for malnutrition^34^ upon arrival in the nursing ward as a part of standard admission procedures. Patients gave consent to collect their food trays after meal consumption after information concerning the project was given orally. Patients were included if they were screened as malnourished (MUST score 2) or at risk for malnutrition (MUST score 1) or if they were indicated as at risk for malnutrition by a dietitian for various reasons (ie, recent weight loss, having nutrition support at home, or reporting low food intake during hospitalization). Age, gender, body mass index, reason for admission, and LOS were recorded. Patients were excluded if they received exclusive or supplemental parenteral or enteral nutrition or if their hospital stay was expected to be <3 days. There was no additional burden on the patients during hospitalization. Retrospective, blinded patient data and observational food intake data were collected under the Agreement on Medical Treatment Act and the Personal Data Protection Act, according to medical ethical standards. The study was registered at www.trialregister.nl (no. NTR6178).

### Provision of Hospital Meals

There were 3 strict time slots every day during which hospital meals were provided: breakfast (∼8:00 am), lunch (∼12:00 pm), and dinner (∼5:30 pm). Patients were provided with voluntary hot and/or cold drinks, snacks, and ONS 3 times a day in between the main meals (at set time points at 10:00 am, 2:00 pm, and 7:00 pm). During the provision of all meals, there was mealtime assistance. Patients chose their main meal and portion size (0.5, 1, or 2) the day before. Data were collected for a minimum of 3 days and data collection was stopped after a maximum 7 days (Supplementary Figure 1).

### Consumption of Hospital Meals

Researchers collected a description of the patients’ ordered meal, which was available on the serving tray. This was done for breakfast, lunch, and dinner. The researchers collected the serving tray, and all leftovers were weighed using a scale (Soehnle, Backnang, Germany) when patients were finished eating. To assess snack and ONS consumption, snack and supplement leftovers were weighed, and the researcher collected wrappers and nursing notes and communicated with food assistants, patients, and family. A researcher was present at the ward during the entire assessment period.

### Oral Nutritional Supplements

Of the total group of 102 patients, *n* = 62 were prescribed with ONS. Energy and/or protein ONS were provided as a cold beverage in between the main meals. To allow assessment of the data based on ONS use, data are presented for the non‐ONS group and ONS group. ONS provision varied from 1 supplement per week to 3 supplements per day, which was a result of the prescription by the dietitian and provision by the food assistant. A variety of different flavors and energy and/or protein ONS were available during the assessment period (for a full description of the prescribed ONS, see Supplementary Table 1).

### Nutrition Content of Hospital Meals

For all provided and consumed food, total energy (MJ and kcal), protein (g and percentage of energy provided by macronutrient [En%]), carbohydrate (g and En%), and fat (g and En%) were calculated, based upon product specifications provided by the food suppliers and the Dutch Food Consumption Database 2016 (NEVO; RIVM, Bilthoven, the Netherlands^35^). The Harris and Benedict equation was used to estimate patients’ basal energy requirements.^36^

### Statistical Analysis

Data were checked for normality; all data were non‐normally distributed, except for energy consumption and protein consumption at dinner. Data are expressed as mean ± SD or median [interquartile range] when appropriate. Food provision and consumption data are expressed as median [interquartile range] (for consistency; both normal and non‐normally distributed data). Differences between provided and consumed food intake were analyzed using Wilcoxon signed rank tests. Mann‐Whitney *U* tests were used to test for differences between the non‐ONS group and ONS group. Spearman ρ test was used to calculate the relationship between daily energy and protein intake. Statistical significance was set at *P* < .05. All calculations were performed using the statistical software program SPSS (version 25.0, IBM Corp, Armonk, NY, USA).

## Results

### Patients’ Characteristics

In total, 102 patients were monitored (males/females, 53/49; age, 68 ± 14 years; LOS, 14 [8–28] days) during hospitalization. In Table 1, patients’ characteristics are presented. Calculated resting metabolic rate averaged 5.61 [5.11–6.44] MJ/d (1340 [1220–1540] kcal/d). In total, 62 patients were prescribed with ONS (ONS group) and 40 patients did not receive ONS (non‐ONS group). The ONS group had a longer hospital stay (18 [11–35] days) than the non‐ONS group (10 [6–17] days) (*P <* .001).

**Table 1:** Patients’ Characteristics

Characteristics	All patients (*n* = 102)	Non‐ONS group (*n* = 40)	ONS group (*n* = 62)
Age, y	68 ± 14	67 ± 15	69 ± 14
Gender (M/F), *n*	53/49	20/20	33/29
Body weight, kg	69.0 ± 16.0	70.8 ± 17.6	67.8 ± 14.9
Height, m	1.71 ± 0.10	1.71 ± 0.10	1.71 ± 0.09
BMI, kg/m^2^	23.6 ± 4.8	24.2 ± 5.2	23.1 ± 4.4
Length of stay, d	14 [8–28]	10 [6–17]^a^	18 [11–35]
Acute admission, *n*	77	25	52
Elective admission, *n*	25	15	10
Respiratory/geriatric/general surgery ward, *n*	36/32/34	11/14/15	25/18/19
Resting metabolic rate, MJ/d/kcal/d	5.61 [5.11–6.44]/1340 [1220–1540]	5.66 [5.22–6.63]/1350 [1250–1580]	5.55 [5.05–6.39]/1320 [1210–1530]

Values are mean ± SD or median [interquartile range] where appropriate.

Resting metabolic rate was calculated based upon the adjusted Harris and Benedict equation.^36^

BMI, body mass index; F, female; M, male; ONS, oral nutritional supplements.

a Significant difference between non‐ONS and ONS group, *P* < .05.

### Energy Provision and Consumption

Hospital meals provided 7.87 [5.82–9.21] MJ/d (1880 [1390–2200] kcal/d) during the entire hospital stay, and energy consumption averaged 4.98 [3.05–6.41] MJ/d (1190 [730–1530] kcal/d). On average, 37% of the provided energy content in hospital meals and snacks was not consumed and was discarded (*P* < .001). Total macronutrient composition of the consumed hospital meals (g and En%) is presented in Table 2.

**Table 2:** Macronutrient Intake in Hospitalized Patients (*n* = 102)

Meal	Energy (MJ/kcal)		Carbohydrate (g and En%)		Protein (g and En%)		Fat (g and En%)	
	Non‐ONS	ONS	Non‐ONS	ONS	Non‐ONS	ONS	Non‐ONS	ONS
Breakfast	0.96 [0.65–1.25]/230 [150–300]	1.30 [0.75–1.69]/310 [180–400]	26 [19–37] (49)	36 [22–45] (47)	8 [6–13] (16)	12 [6–17] (15)	8 [5–13] (33)	13 [7–18] (36)
Lunch	1.04 [0.60–1.53]/250 [140–370]	1.16 [0.55–1.69]/280 [130–400]	31 [19–46] (50)	35 [16–46] (49)	8 [5–14] (14)	10 [5–16] (14)	9 [4–16] (34)	10 [5–17] (34)
Dinner	0.99 [0.62–1.52]/240 [150–360]	1.23 [0.91–1.65]/300 [220–390]	28 [19–49] (52)	40 [28–51] (53)	11 [7–17] (20)	13 [9–18] (17)	7 [4–12] (26)	9 [5–13] (28)
Snacks	0.56 [0.36–0.76]/130 [90–180]	0.56 [0.25–0.86]/130 [60–200]	25 [17–39] (75)	24 [9–35] (72)	1 [0–1] (8)	1 [0–4] (8)	1 [0–4] (17)	2 [0–5] (27)
ONS		1.26 [0.40–1.79]/300 [100–430]		37 [13–55] (55)		11 [4–16] (16)		9 [3–15] (31)

Values are expressed as median [interquartile range].

ONS was prescribed in *n* = 62.

En%, percentage of energy provided by macronutrient; ONS, oral nutritional supplements.

### Protein Provision and Consumption

**Figure 1:**
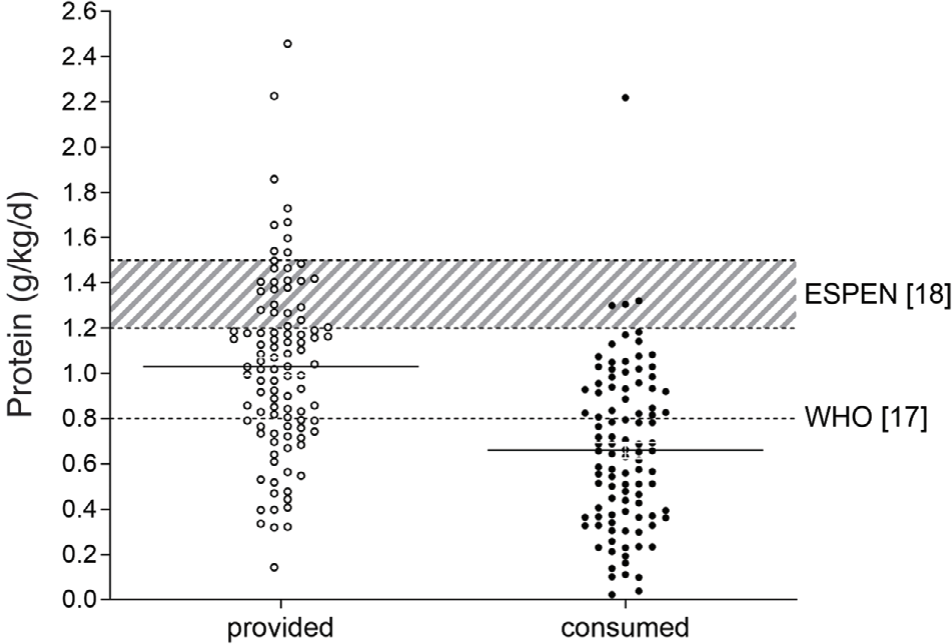
Protein provision and consumption (g/kg/d) during hospitalization. Protein intake was measured in *n* = 102 patients over, on average, 4.6 hospitalization days. The dotted line represents the recommended protein intake of 0.8 g/kg/d suggested for healthy adults of all ages by the World Health Organization (WHO).^17^ The shaded area represents the recommended protein intake of 1.2–1.5 g/kg/d suggested for malnourished patients or patients at risk for malnutrition due to acute or chronic illness.^18^ ESPEN, European Society for Clinical Nutrition and Metabolism.

Protein provision and consumption (g/kg/d) from self‐selected hospital meals, snacks, and ONS during hospitalization are presented in Figure 1. Protein provision was 1.03 [0.77–1.26] g/kg/d during hospitalization, whereas actual protein consumption was 0.65 [0.37–0.93] g/kg/d. The consumed amount of protein was 37% (0.38 of 1.03 g/kg/d) lower than the provided amount of protein (*P* < .001). Only 4% (4 of 102) of the patients had a protein intake equal to or above recommended protein guidelines (≥1.2 g/kg/d^1^). Thirty‐five percent (36 of 102) of the patients met the protein requirements set by WHO (≥0.8 g/kg/d).

### Energy Provision and Consumption Between Groups

**Figure 2:**
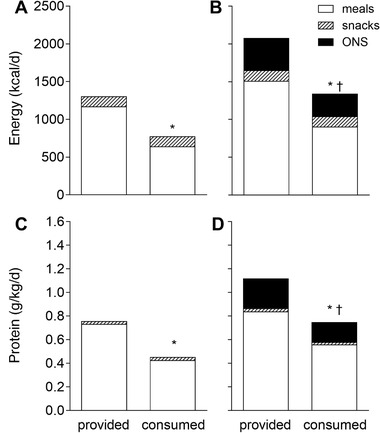
Total dietary energy provision and consumption (kcal/d) (A and B) and protein provision and consumption (g/kg/d) (C and D) across main meals in patients during hospitalization. Total energy and protein intake in patients that were not prescribed with oral nutritional supplements (non‐ONS; A and C; *n* = 40) and patients that were prescribed with ONS (B and D; *n* = 62). *Significant difference when compared with provided, *P* < .001. ^†^Significant difference when compared with non‐ONS group, *P* < .001.

Energy provision and consumption from self‐selected hospital meals, snacks, and ONS during hospitalization are presented in Figure 2A and 2B. Hospital meals provided 5.83 [4.65–7.54] MJ/d (1390 [1110–1800] kcal/d) and 8.82 [7.51–9.95] MJ/d (2110 [1790–2380] kcal·d^−1^) during the entire hospitalization period in the non‐ONS (Figure 2A) and ONS (Figure 2B) group, respectively. Energy consumption was 3.22 [2.73–5.27] MJ/d (770 [650–1260] kcal/d) vs 5.67 [3.67–7.15] MJ/d (1350 [880–1710] kcal/d), respectively. On average, 37% of the provided energy content was not consumed and was discarded (*P* < .001). After correction for ONS consumption in the ONS group, there were no differences in total energy intake consumed from meals and snacks between the non‐ONS and ONS groups.

### Protein Provision and Consumption Between Groups

Protein provision and consumption (g/kg/d) from self‐selected hospital meals, snacks, and ONS during hospitalization are presented in Figure 2C and 2D. Main meals provided 0.73 [0.53–1.00] g/kg/d protein in the non‐ONS group (Figure 2C) and 0.84 [0.68–1.09] g/kg/d protein in the ONS group (Figure 2D). Snacks provided merely 0.03 [0.01–0.04] and 0.03 [0.01–0.07] g/kg/d protein in the non‐ONS and ONS group, respectively. ONS added 0.25 [0.14–0.34] g/kg/d protein to total protein provision. Protein consumption through the consumption of main meals and snacks in the non‐ONS group was 0.43 [0.31–0.65] and 0.02 [0.01–0.04] g/kg/d, respectively, whereas protein consumption in the ONS group was 0.56 [0.38–0.72], 0.02 [0.01–0.05], and 0.17 [0.05–0.25] g/kg/d from the consumption of main meals, snacks, and ONS, respectively. On average, 32% (0.08 of 0.25 g/kg/d) of the ONS provided was not consumed. Patients in the ONS group had a higher total protein intake when compared with patients in the non‐ONS group (*P* < .001). Protein intake from the consumed hospital meals and snacks did not differ between groups when ONS contribution was excluded in the ONS group (*P* = .169).

### Protein Intake Distribution

**Figure 3:**
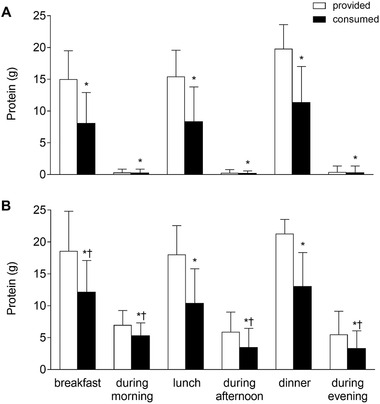
Median [interquartile range] dietary protein provision and consumption (g) across main meals in patients in the non‐ONS group (A; *n* = 40) and ONS group (B; *n* = 62) during hospitalization. *Significant difference when compared with provided, *P* < .001. ^†^Significant difference when compared with non‐ONS group, *P* < .05. ONS, oral nutritional supplement.

Distribution of protein provision and consumption (g) across main meals is presented in Figure 3. Hospital meals provided 15 [11–19], 15 [12–20], and 20 [15–24] g protein in the non‐ONS group (Figure 3A) and 19 [14–25], 18 [13–23], and 21 [19–24] g protein in the ONS group (Figure 3B) at breakfast, lunch, and dinner, respectively. Protein consumption was 35%, 36%, and 34% less than protein provided at breakfast, lunch, and dinner, respectively (all *P* < .001). As a result, actual protein intake was 8 [6–13], 8 [5–14], and 11 [7–17] g in the non‐ONS group and 12 [6–17], 10 [5–16], and 13 [9–18] g in the ONS group at breakfast, lunch, and dinner, respectively. Protein intake at breakfast was higher in the ONS group when compared with the non‐ONS group (*P* = .044). Snacks contributed only 0.3 [0.1–0.9] g protein in the morning, afternoon, and evening in the non‐ONS group. In the ONS group, snacks and ONS contributed 5 [2–7], 4 [2–6], and 3 [1–6] g protein to total protein intake during the morning, afternoon, and evening, respectively.

**Figure 4:**
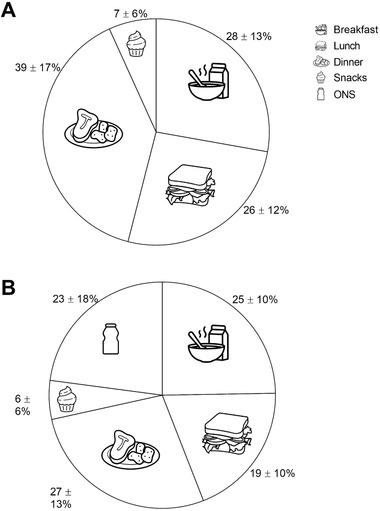
Distribution of protein intake across main meals, snacks, and ONS (expressed as a percentage of the total amount of protein consumed) during hospitalization in non‐ONS group (A; *n* = 40) and ONS group (B; *n* = 62). ONS, oral nutritional supplements.

Protein intake distribution per main meal expressed as the percentage of the total amount of protein consumed is presented in Figure 4. Breakfast, lunch, and dinner provided 28% ± 13%, 26% ± 12%, and 39% ± 17% and snacks contributed 7% ± 6% of total daily protein intake in the non‐ONS group (Figure 4A). In the ONS group, breakfast, lunch, and dinner provided 25% ± 10%, 19% ± 10%, and 27% ± 13% of the daily protein intake. Snacks contributed 6% ± 6% of daily protein intake and ONSs contributed the remaining 23% ± 18% of daily protein intake in the ONS group (Figure 4B).

**Figure 5:**
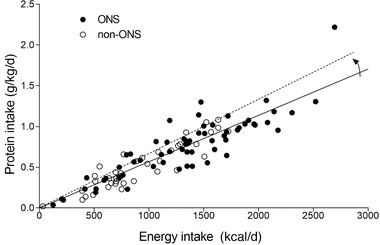
Daily energy and total protein intake in older patients at risk for malnutrition during hospitalization. The dotted line represents the association line between energy and protein intake if a more protein‐dense diet were provided. ONS, oral nutritional supplements.

Dietary protein intake strongly correlated with daily energy intake in both the non‐ONS group (*r* = 0.894; *P* < .001) and ONS group (*r* = 0.860; *P* < .001) (Figure 5). Relative contribution of protein to total energy intake (En% protein) did not differ between groups (*P* = .422).

## Discussion

Despite a protein provision of 1.0 g/kg/d, protein intake was merely 0.65 kg/kg/d in older patients who were deemed at risk for malnutrition during their hospitalization. In total, 37% of the provided food was discarded and 32% of the provided ONS were not consumed. Total energy and protein intake per day were greater in those patients receiving ONS, which did not affect the macronutrient composition of the diet. Median protein intake per main meal ranged from 8 to 13 g protein in all patients.

In the present study, we assessed the consumption of self‐selected hospital meals, snacks, and ONS in patients who were classified as at risk for malnutrition during their hospitalization. Daily energy intake was merely 5.0 MJ/d (1200 kcal/d), which is well below patients’ calculated resting metabolic rate (5.6 MJ/d/1340 kcal/d). Consequently, patients seemed to remain in a negative energy balance during their entire hospital stay. An energy deficit during hospitalization accelerates the loss of skeletal muscle mass and strength.^37^ Muscle atrophy typically observed during hospitalization^14^, ^15^ has been attributed to the lack of sufficient protein consumed as a direct consequence of a decline in food intake. Current guidelines suggest a protein intake of 1.2–1.5 g/kg/d to support muscle mass maintenance in malnourished patients or patients at risk for malnutrition due to acute or chronic illness.^18^, ^19^ In the present study, we show that protein consumption during long‐term hospitalization does not even nearly reach the suggested protein intake guidelines. In fact, <4% of the patients consumed ≥1.2 g/kg/d protein. Merely 35% of the patients consumed an amount of protein that was equal to or more than the required 0.8 g/kg/d that is prescribed by WHO for healthy adults (Figure 1).

The amount of protein that was provided via the hospital diet was 1.0 g/kg/d. Even if patients would have fully consumed all meals and ONS that were provided, the ESPEN guidelines on daily protein intake would not have been reached in 72% of the patients. With 30%–40% of the provided food and supplements being discarded, daily protein intake did not even reach WHO guidelines on recommended protein intake. In our hospital, current practices to increase protein intake in these patients include the provision of energy and/or protein‐rich snacks in between main meals, counseling to motivate patients to choose protein‐rich products, and/or the prescription of ONS. Clearly though, these existing strategies are not effective enough to reach a protein intake at the level of WHO, let alone the ESPEN guidelines on protein intake for patients. Moreover, during hospitalization, patients are less physically active and food intake is typically reduced. To maintain protein intake at habitual intake levels under conditions of a reduced energy intake likely requires the installment of a more protein‐dense diet.

One of the often applied strategies to increase protein intake is to provide ONS. The current study was an observational study. We assessed how many ONS a patient received and/or consumed during their hospital stay. We noticed that even when the dietitian prescribed multiple supplements per week, supplements were often not provided or not consumed by the patients. This resulted with some patients consuming only 1 supplement per week, which would not likely have any impact. Previous work showed higher energy and protein intake in patients being prescribed with ONS, providing up to 8–30 g protein extra per day.^31^, ^38^, ^39^ Our data extend these findings by showing that even though ∼30% of the ONS were discarded, they still seemed effective in increasing energy and protein intake when compared with those patients not receiving ONS. However, providing patients with ONS did not change the protein density of the hospital diet (Figure 5). Though ONS provision did increase absolute daily energy and protein intake, the increase in protein intake would be greater if products with greater protein content were used. Using more protein‐dense ONS may represent an effective strategy to allow the diet to become more protein dense, with relatively more protein being consumed at the same or even a lower energy intake level (Figure 5). This is especially required to allow patients to maintain their habitual protein intake level, which is necessary to attenuate muscle mass loss during hospitalization. As there are numerous types of ONS with varying protein contents for different patient populations, dietitians should make an informed decision on the optimal prescription of the right product(s) and matching diet for each individual patient.

Previous research has shown that to increase postprandial muscle protein synthesis rates, ingestion of at least 20 g of a high‐quality protein is needed.^40^, ^41^, ^42^, ^43^, ^44^ Our data clearly show that the amount of protein consumed at breakfast (10 [6–15] g), lunch (9 [5–14] g), and dinner (13 [9–18] g) remains well below the proposed 20 g (Figure 3). As this is in line with previous work showing inadequate protein intake with breakfast and lunch, it is essential to increase the protein content of each meal.^20^, ^45^, ^46^, ^47^ Apart from using protein‐dense ONS as described above, providing more protein‐dense products or fortifying main meals would allow patients to consume more protein per meal. Previous studies have shown that providing protein‐fortified foods (such as bread, yogurt, cake, fruit juice, and soup) or using more protein‐dense foods (such as dairy, eggs, fish, meat) can be effective in increasing both absolute as well as relative protein intake during hospitalization.^21^, ^29^, ^30^ Another potential strategy to increase total daily protein intake could be by creating an additional meal moment to consume a protein‐rich snack or supplement. The ingestion of a protein‐rich snack before sleep could serve as such an additional meal moment to increase protein intake. Our laboratory has recently shown that protein ingestion prior to sleep increases overnight muscle protein synthesis rates in older individuals.^48^, ^49^ Whether these nutrition intervention strategies are effective to increase daily protein and/or energy intake during hospitalization remains to be assessed.

In conclusion, energy and protein intake levels are well below suggested guidelines in hospitalized patients at risk for malnutrition. As 30%–40% of the provided food and supplements are not consumed, actual protein consumption remains well below the minimal requirements of 0.8 g/kg/d and far below recommended intake levels of 1.2–1.5 g/kg/d. Although the provision of ONS increases habitual energy and protein intake, it does not affect the macronutrient composition of the diet. Current nutrition strategies to achieve the recommended daily protein intake in older patients during their hospitalization are not as effective as generally assumed and should be redesigned.

## Statement of Authorship

M. E. G. Weijzen, I. W. K. Kouw, and L. J. C. van Loon equally contributed to the conception and design of the research; P. Geerlings contributed to the design of the research; M. E. G. Weijzen contributed to the acquisition and analysis of the data; M. E. G. Weijzen, I. W. K. Kouw, P. Geerlings, L. B. Verdijk, and L. J. C. van Loon contributed to the interpretation of the data; and M. E. G. Weijzen, I. W. K. Kouw, and L. J. C. van Loon drafted the manuscript. All authors critically revised the manuscript, agree to be fully accountable for ensuring the integrity and accuracy of the work, and read and approved the final manuscript.

## Supporting information

Supporting information.

Supporting information.

Supporting information.
