# Supplementary figures and images for: During Hospitalization, Older Patients at Risk for Malnutrition Consume <0.65 Grams of Protein per Kilogram Body Weight per Day
Source: Nutr Clin Pract. 2020 Jun 24;35(4):655–63. doi: 10.1002/ncp.10542 (PMC7384011; doi:10.1002/ncp.10542)

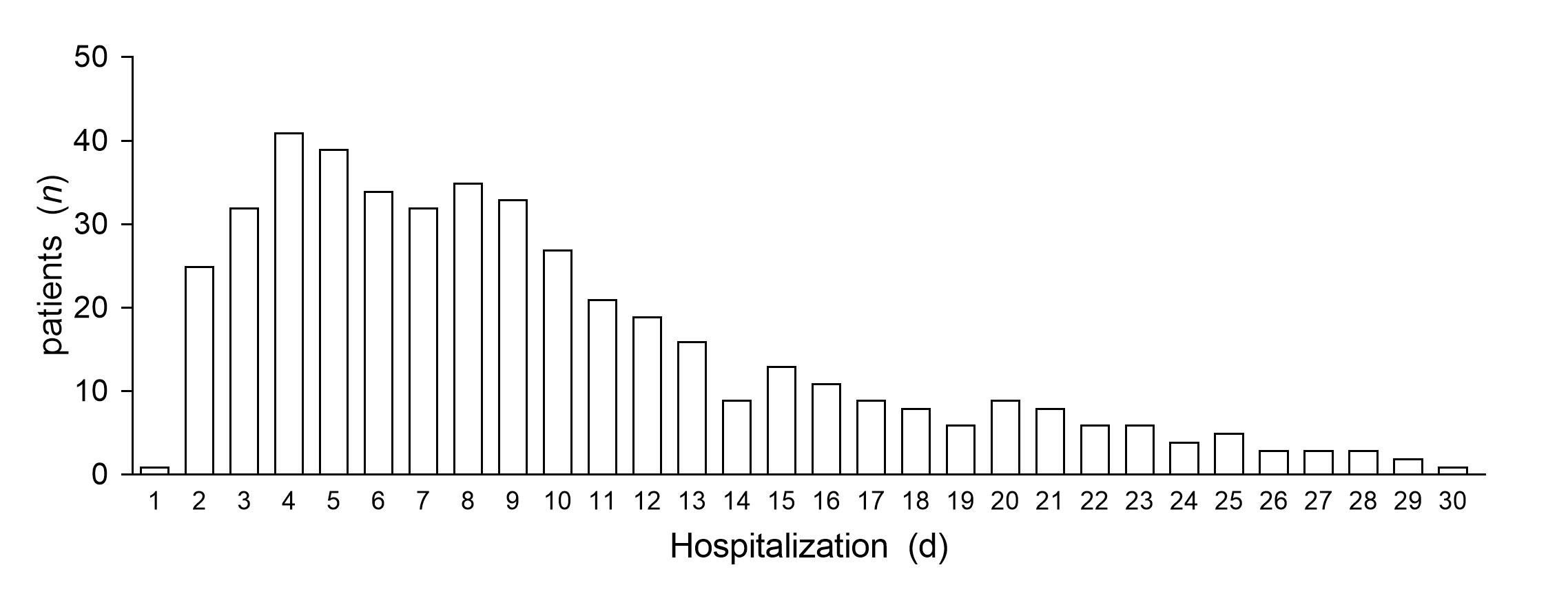

Supplement: Supplementary file 1 — Supporting information. [file NCP-35-655-s001.jpg]
