# Supplementary material for: During Hospitalization, Older Patients at Risk for Malnutrition Consume <0.65 Grams of Protein per Kilogram Body Weight per Day
Source: Nutr Clin Pract. 2020 Jun 24;35(4):655–63. doi: 10.1002/ncp.10542 (PMC7384011; doi:10.1002/ncp.10542)
Supplement: Supplementary file 2 — Supporting information. [file NCP-35-655-s002.docx]

**Table S1.** Macronutrient composition of oral nutritional supplements per serving

|  | Volume (ml) | Energy (MJ/kcal) | Carbohydrate (g and En%) | Protein  (g and En%) | Fat (g and En%) |
| --- | --- | --- | --- | --- | --- |
| Nutridrink compact | 125 | 1260/300 | 37 (50) | 12 (16) | 12 (35) |
| Nutridrink compact Multifibre | 125 | 1256/300 | 32 (42) | 12 (16) | 13 (39) |
| Nutridrink | 200 | 1250/300 | 37 (49) | 12 (16) | 12 (35) |
| Nutridrink Multifibre | 200 | 1260/300 | 37 (49) | 12 (16) | 12 (35) |
| Nutridrink Juicy style | 200 | 1270/300 | 67 (89) | 8 (10) | 0 (0) |
| Nutridrink protein | 200 | 1260/300 | 33 (45) | 18 (24) | 10 (31) |
| Renilon 4.0 | 125 | 1044/250 | 29 (47) | 5 (8) | 13 (45) |
| Renilon 7.5 | 125 | 1044/250 | 25 (40) | 9 (15) | 13 (45) |

**LEGEND**

**Figure S1**. Patients (*n*) included and followed during their days of hospitalization. Patients were followed for a minimum of three hospitalization days and data collection was stopped after seven days.
