# Supplementary material for: During Hospitalization, Older Patients at Risk for Malnutrition Consume <0.65 Grams of Protein per Kilogram Body Weight per Day
Source: Nutr Clin Pract. 2020 Jun 24;35(4):655–63. doi: 10.1002/ncp.10542 (PMC7384011; doi:10.1002/ncp.10542)
Supplement: Supplementary file 3 — Supporting information. [file NCP-35-655-s003.docx]

**Table S2.** Product options of generic hospital menu

| *Breakfast / Lunch* | | | |
| --- | --- | --- | --- |
| ***Bread*** | ***Toppings*** | ***Dairy*** | ***Other*** |
| White bread | Butter | Semi-skimmed milk | Fruit juice |
| Brown bread | Smoked beef | Buttermilk | Fruit |
| Whole grain bread | Chicken breast | Semi -skimmed Chocolate milk | Tomato salad |
| Rye bread | Ham | Low-fat yoghurt | Fruit salad |
| Rusk | Cervelat | Fruit yoghurt | Instant soup |
| Ginger bread | Pate | Curd cheese | Boiled egg |
| Crispbread | Cheese | Vanilla custard | Coffee |
| Cornflakes | Cumin cheese | Chocolate custard | Tea |
| Muesli | Herb cheese |  |  |
|  | Marmalade |  |  |
|  | Chocolate sprinkles |  |  |
|  | Chocolate spread |  |  |
|  | Peanut butter |  |  |
|  | Syrup |  |  |
|  | | | |
| *Dinner* | | | |
| ***Meat/ gravy*** | ***Starch products*** | ***Vegetables*** | ***Dessert*** |
| Baked fish | Pommes duchesse | Peas, carrots and corn | Vanilla and chocolate custard |
| Chicken breast | Boiled potatoes | Peas and carrots | Vanilla yoghurt |
| Turkey steak | Mashed potatoes | Spinach | Forest fruit custard cheese |
| Beef steak | Rice | Green beans | Low-fat yoghurt |
| Veal meatball | Macaroni | Beetroot | Apricot compote |
| Ham steak |  | Cauliflower |  |
| Poached fish |  | Coleslaw |  |
| Omelet |  | Apple sauce |  |
| Vegetable spring roll |  |  |  |
| Gravy |  |  |  |
